# Thrombopoietin Receptor Agonist Mitigates Hematopoietic Radiation Syndrome and Improves Survival after Whole-Body Ionizing Irradiation Followed by Wound Trauma

**DOI:** 10.1155/2017/7582079

**Published:** 2017-03-20

**Authors:** Juliann G. Kiang, Min Zhai, Pei-Jun Liao, Connie Ho, Nikolai V. Gorbunov, Thomas B. Elliott

**Affiliations:** ^1^Radiation Combined Injury Program, Armed Forces Radiobiology Research Institute, Bethesda, MD 20889, USA; ^2^Department of Pharmacology and Molecular Therapeutics, Uniformed Services University of the Health Sciences, Bethesda, MD 20814, USA; ^3^Department of Medicine, Uniformed Services University of the Health Sciences, Bethesda, MD 20814, USA; ^4^College of Letters & Science, University of California, Berkeley, CA 94510, USA

## Abstract

Ionizing radiation combined with trauma tissue injury (combined injury, CI) results in greater mortality and H-ARS than radiation alone (radiation injury, RI), which includes thrombocytopenia. The aim of this study was to determine whether increases in numbers of thrombocytes would improve survival and mitigate H-ARS after CI. We observed in mice that WBC and platelets remained very low in surviving RI animals that were given 9.5 Gy ^60^Co-*γ*-photon radiation, whereas only lymphocytes and basophils remained low in surviving CI mice that were irradiated and then given skin wounds. Numbers of RBC and platelets, hemoglobin concentrations, and hematocrit values remained low in surviving RI and CI mice. CI induced 30-day mortality higher than RI. Radiation delayed wound healing by approximately 14 days. Treatment with a thrombopoietin receptor agonist, Alxn4100TPO, after CI improved survival, mitigated body-weight loss, and reduced water consumption. Though this therapy delayed wound-healing rate more than in vehicle groups, it greatly increased numbers of platelets in sham, wounded, RI, and CI mice; it significantly mitigated decreases in WBC, spleen weights, and splenocytes in CI mice and decreases in RBC, hemoglobin, hematocrit values, and splenocytes and splenomegaly in RI mice. The results suggest that Alxn4100TPO is effective in mitigating CI.

## 1. Introduction

Detonation of radiation dispersal devices or nuclear weapons is expected to induce ionizing radiation injury (RI) or combined injury (CI) (i.e., RI in combination with trauma from blast, thermal energy exposure, wound, hemorrhage, etc.). RI induces DNA double-strand breaks* in vivo* [1] and* in vitro* [2, 3], activates NF-*κ*B/NF-IL6/iNOS signal transduction pathways, elevates IL-6, IL-10, KC, G-CSF, and MCP-1 cytokine/chemokine concentrations in the peripheral blood, and increases systemic bacterial infection, thereby leading to cell death and multiple organ dysfunction (MOD) and failure (MOF) [1, 4–7]. CI amplifies RI-induced acute radiation syndrome (ARS), thereby increasing mortality [1, 5–7]. Because the ARS induced by RI and CI occurs at several biological, molecular, cellular, tissue, and systemic levels, the complexity of the responses makes it particularly difficult to evaluate and identify countermeasures for prophylaxis, mitigation, or therapy.

Hematopoietic tissue is highly susceptible to cytotoxic effects of ionizing radiation, resulting in thrombocytopenia and/or neutropenia and lymphopenia, followed by mortality [7]. RI remarkably decreases the number of platelets in mouse blood 7 days postirradiation, and, at that same time point, CI further decreases platelet numbers in mouse blood [1, 8]. The decrease was not fully recovered even 30 days postirradiation, although the CI-induced augmentation is no longer present at this time point [9, 10]. Thrombopoietin (TPO) has been used to prevent thrombocytopenia, accelerate platelet and RBC reconstitution, alleviate neutropenia, and promote the recovery of immune bone marrow cells [11, 12]. Small molecular-weight TPO mimetics such as Alxn4100TPO can enhance platelet numbers [13]. Alxn4100TPO is a short chain peptide incorporated in both light and heavy chains of human immunoglobulin G. In CD2F1 mice, 75% survived when 1 mg/kg dose of the drug was given at s.c. 12 h postirradiation following 9 Gy ^60^Co gamma-photon radiation at 0.6 Gy/min [13]. The half-life of Alxn4100TPO in serum is 13 days [14].

We hypothesized that treatment with TPO mimetic would demonstrate therapeutic effects for RI and/or CI. Herein, we evaluated parameters of survival, body weight, wound healing, numbers of platelets, red blood cells and white blood cells, spleen mass, and numbers of splenocytes after treatment with Alxn4100TPO administered 24 h after RI and CI in combination with gentamicin and levofloxacin antimicrobial therapy to prevent sepsis in an established B6D2F1/J mouse model by Ledney et al. [5, 15].

## 2. Materials and Methods

Research was conducted in a facility accredited by the Association for Assessment and Accreditation of Laboratory Animal Care (AAALAC). All procedures involving animals were reviewed and approved by the AFRRI Institutional Animal Care and Use Committee. Euthanasia was carried out in accordance with the recommendations and guidance of the American Veterinary Medical Association [16, 17].

### 2.1. Animals and Experimental Design

B6D2F1/J female mice (12 weeks old) obtained from the Jackson Laboratory (Bar Harbor, ME) were maintained in a facility accredited by AAALAC in plastic microisolator cages on hardwood chip bedding and allowed to acclimate to their surroundings for at least 14 days prior to initiation of the study. Male mice were not used in this study because of problems associated with male mouse aggression, which in these experiments could lead to further damage to wound sites and enhanced infection. All mice were randomly assigned to experimental groups. They were randomly divided to different experimental groups. For platelet time-course study, animals were not treated with vehicle or Alxn4100TPO (*N* = 6 per group and per each time point). For hematology analysis, each group had *N* = 6. For survival experiment, they were vehicle treatment groups, sham (*N* = 20), wound (*N* = 19), radiation alone (RI, *N* = 22), and radiation combined with wound (CI, *N* = 22), and Alxn4100TPO treatment groups, sham (*N* = 20), wound (*N* = 20), RI (*N* = 22), and CI (*N* = 22). Commercial rodent chow and acidified tap water were provided* ad libitum*. Animal holding rooms were maintained at 21°C ± 1°C with 50%  ± 10% relative humidity using at least 10 changes/h of 100% conditioned fresh air. A 12 h 0600 (light) to 1800 (dark) full-spectrum lighting cycle was used. The AFRRI Institutional Animal Care and Use Committee approved all animal procedures.

### 2.2. Gamma Irradiation

Mice were restrained in well-ventilated acrylic plastic boxes (4 mice per box) and given a dose of 9.5 Gy gamma photons in a bilateral radiation field at a dose rate of 40 cGy/min in the AFRRI cobalt-60 gamma-irradiation facility [8]. Eight mice were housed per filter-topped polycarbonate cage (MicroIsolator) in conventional holding rooms. The alanine/ESR (electron spin resonance) dosimetry system (American Society for Testing and Material Standard E 1607) was used to measure dose rates to water in cores of acrylic mouse phantoms. The ESR signals were measured against a calibration curve based on standard calibration dosimeters provided by the US National Institute of Standards and Technology [18]. The accuracy of the calibration curves was verified by intercomparison with the National Physical Laboratory (United Kingdom). The radiation field was uniform within ±2%. Sham-treated mice were placed in boxes, transported to the irradiation facility, where they were held for approximately 10 min and returned to the veterinary facility.

### 2.3. Skin Injury

Full-thickness skin injuries were inflicted on the shaved dorsal surface of the mice as previously described [5, 6]. Animals receiving skin wounds were anesthetized by isoflurane inhalation and a 15% total body surface area (TBSA) skin wound was inflicted within 1 h after irradiation with a procedure described elsewhere [5, 6]. Briefly, 24 h prior to experiments, hair of the dorsal surface of mice was removed under anesthesia (isoflurane inhalation) using electric clippers. Within 1 h after irradiation or sham irradiation, mice were anesthetized by isoflurane inhalation, and wounding or sham wounding was performed. A nonlethal total body surface area (TBSA) wound was administered 19 ± 1.3 mm from the occipital bone and between the scapulae using a stainless steel punch on a Teflon-covered board cleaned with 70% alcohol before each use. The panniculus carnosus muscle and overlying skin (23.5 ± 1.1 mm in length and 14.9 ± 0.7 mm in width) were removed [7]. For sham-treated animals, mice received the same procedure except without actual skin wounding.

Skin-wounded mice in wounded groups and CI groups received one dose of 150 mg/kg of acetaminophen (for analgesia) in 0.5 mL 0.9% sodium chloride solution immediately after skin injury, but sham-treated animals and irradiated alone animals received one dose of only 0.5 mL 0.9% sodium chloride solution immediately after a sham skin procedure.

### 2.4. Alxn4100TPO

This drug was obtained from Alexion Pharmaceuticals Inc. (Cheshire, Connecticut). Either Alxn4100TPO (1 mg/kg, s.c.) or vehicle (sterile 0.9% sodium chloride solution for injection, USP; 0.2 ml/mouse s.c.) was injected once into dorsal scruff of neck of mice without anesthesia 24 h after sham, wounding, RI, or CI. The drug dose was selected based on the previous publication [13] in that this drug at 1 mg/kg with one injection displayed the best survival outcome. Injection 24 h postirradiation [10] was selected to assure sufficient time for patients or accident victims to get to nearby clinical settings in order to receive professional care.

### 2.5. Antimicrobial Agents

Gentamicin sulfate cream, 0.1% (generic, E. Fougera and Co., Melville, NY, NDC 0168-007-15), was applied daily for 10 days to the skin injuries on days 1–10. Levofloxacin (LVX), (generic, Aurobindo Pharma, Ltd., Mahabubnagar, India, NDC 65862-537-50), 100 mg/kg in 0.2 ml/mouse, was administered p.o. daily for 14 days on days 3–16. Briefly, a 500 mg tablet was crushed by mortar and pestle. The LVX in the powder was dissolved in a volume of sterile water approximately one-third the total volume required to prepare the concentration needed for the average body mass of the mice to be treated. The suspension was centrifuged to remove the particulate filler and the supernatant solution was passed through a 0.45 *µ*m membrane filter into a sterile amber bottle, which was sealed with a sterile rubber stopper.

### 2.6. Survival and Body Weight

Animals were monitored at least twice daily for their general health and survival for 30 days. Their body weights were measured on days 0, 1, 3, 7, 14, 21, and 28.

### 2.7. Water Consumption

Water consumption (group mean) was measured daily with graduated bottles containing water until day 7 postirradiation.

### 2.8. Wound Closure

Assessments of wound closure were performed on days 1, 7, 14, 21, and 28. Wounds were measured to within 0.01 mm by a caliper with an electronic digital display. The average area of each wound was calculated as *π*(diameter *A*/2) × (diameter *B*/2), where *π* is 3.1416; *A* and *B* represent diameters at right angles to each other.

### 2.9. Assessment of Blood Cell Profile in Peripheral Blood

Blood samples were collected in EDTA tubes on day 30 after RI or CI. Whole blood (0.7–1 ml) was collected by cardiac puncture from mice anesthetized with isoflurane. Then, blood samples were assessed with the ADVIA 2120 Hematology System (Siemens, Deerfield, IL). Differential analysis was conducted using the peroxidase method and light scattering techniques recommended by the manufacturer.

### 2.10. Measurement of Spleen Weight and Splenocyte Counts

Spleens collected on day 30 were weighed first. They were then placed in a plastic pouch containing 10 ml Hanks' Balanced Saline Solution (HBSS, Gibco, Life Technologies, Inc., Carlsbad, CA). The pouch was inserted into a Stomacher® 80 Biomaster Lab System (Seward, Port Saint Lucie, FL) for 60 sec. The contents were poured into a tube through a 70 *μ*m cell strainer (Falcon, Durham, NC), centrifuged at 3000 rpm [i.e., 1960 rcf] for 10 min. The cell pellet was resuspended in 5 ml ACK Lysing buffer (Gibco, Life Technologies, Inc., Carlsbad, CA) and incubated at 37°C for 5 min. Spleen cells were pelleted at 3000 rpm for 10 min and resuspended in HBSS. Splenocytes were counted using a Countess™ automated cell counter (Invitrogen, Carlsbad, CA).

### 2.11. Statistical Analysis

Parametric data are expressed as the mean ± SEM. For each survival experiment, groups of 20–22 mice were evaluated. Survival analyses were performed using one-way ANOVA. For experiments of platelet time-course studies and hematological analysis (*n* = 6 per group), ANOVA, studentized-range test, and Student's *t*-test were used for comparison of groups, with 5% as a significant level.

## 3. Results

### 3.1. RI and CI Decrease Numbers of Platelets and Alxn4100TPO Mitigates Their Decreases

**Figure 1:**
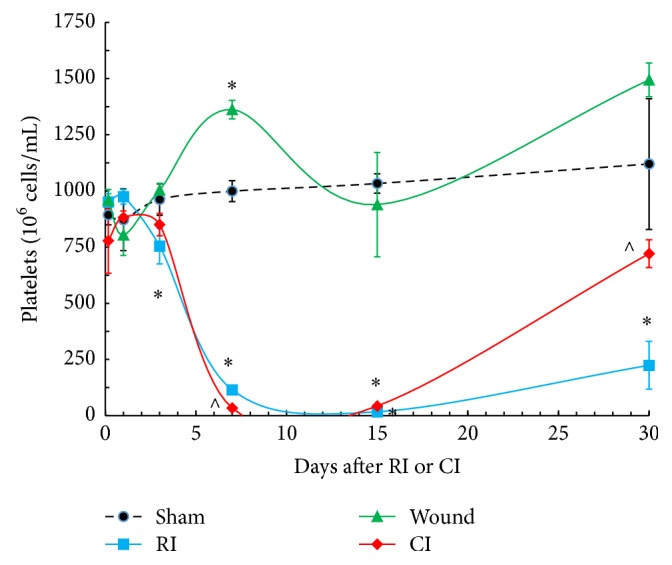
Numbers of platelets were depleted in the first 7 days after RI and CI and then partially recovered gradually between days 15 and 30. Platelet numbers at 4-5 h and days 1, 3, 7, 15, and 30 in surviving sham, wounded, RI, and CI mice without Alxn4100TPO treatment. *N* = 6 per group. Data are presented as mean ± SEM. ^*∗*^*p* < 0.05 versus sham; ^∧^*p* < 0.05 versus RI. CON: control; RI: 9.5 Gy; CI: 9.5 Gy and skin wound.

RI and CI depleted numbers of platelets in peripheral circulation beginning on day 3, which reached their nadir between days 7 and 15 and then recovered gradually as shown previously [8]; whereas skin wounding alone increased platelet counts on day 7 after injury (Figure 1). CI appeared to enhance the RI-induced decrease slightly on day 7. On day 30, platelet numbers recovered faster in CI mice than in RI mice, but did not yet return to the baseline in sham mice by day 30 (Figure 1).

**Figure 2:**
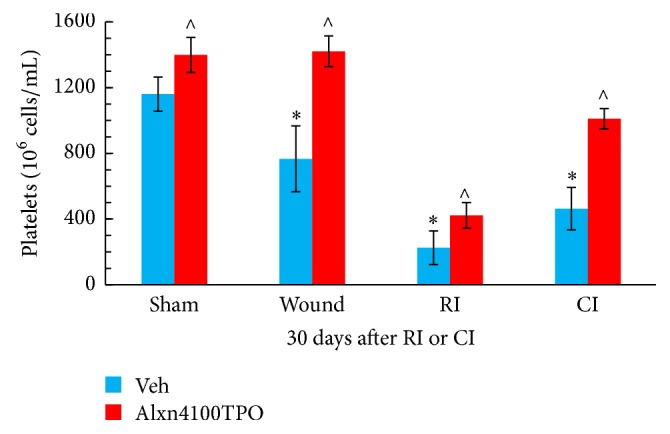
Alxn4100TPO mitigated platelet loss after RI and CI. Platelets were counted on day 30 in surviving sham, wounded, RI, and CI mice. *N* = 6 per group. Data are presented as mean ± SEM. ^*∗*^*p* < 0.05 versus Vehicle Sham; ^∧^*p* < 0.05 versus respective vehicle group. RI: 9.5 Gy; CI: 9.5 Gy and skin wound.

Treatment with Alxn4100TPO significantly mitigated reduction of platelet numbers in RI and CI mice compared to the vehicle-treated RI and CI groups. The Alxn4100TPO-induced increases in platelet counts were 90% and 118% above the vehicle groups, respectively. This treatment also increased platelet counts in sham and wounded mice (Figure 2).

### 3.2. RI and CI Decrease WBC, but Alxn410TPO Mitigates Only the CI-Induced WBC Decreases

**Figure 3:**
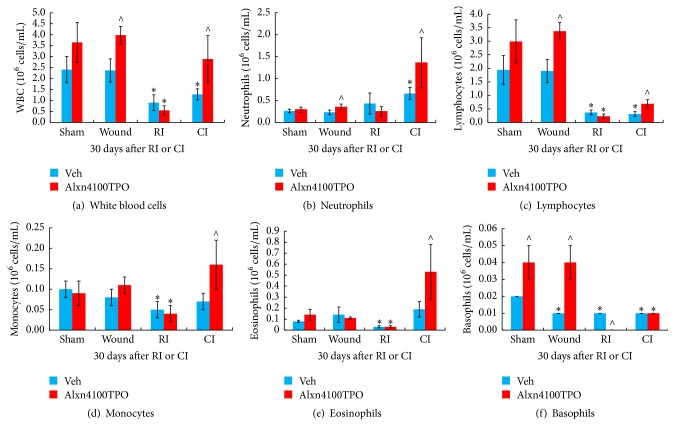
RI and CI decreased WBC, but Alxn4100TPO significantly mitigated only the CI-induced WBC decrease. Numbers of WBC and component cell categories in mouse blood samples collected 30 days after RI and CI were determined. *N* = 4–6 per group. Data are presented as mean ± SEM. ^*∗*^*p* < 0.05 versus Vehicle Sham; ^∧^*p* < 0.05 versus respective vehicle group. RI: 9.5 Gy; CI: 9.5 Gy and skin wound.

RI and CI are known to deplete WBC, whereas skin wounding alone did not affect WBC profiles [1]. In CI surviving mice, Alxn4100TPO significantly mitigated the CI-induced WBC depletion (Figure 3(a)), mainly by increasing numbers of neutrophils (Figure 3(b)), lymphocytes (Figure 3(c)), monocytes (Figure 3(d)), and eosinophils (Figure 3(e)). In contrast, in RI surviving mice, Alxn4100TPO failed to improve WBC profiles (Figure 3). This treatment significantly increased numbers of lymphocytes (Figure 3(c)) and basophile (Figure 3(f)) in wounded mice as well as basophils in sham mice (Figure 3(f)).

### 3.3. RI and CI Decrease RBC, Hemoglobin, and Hematocrit in Blood but Alxn410TPO Mitigates Only the RI-Induced Decreases

**Figure 4:**
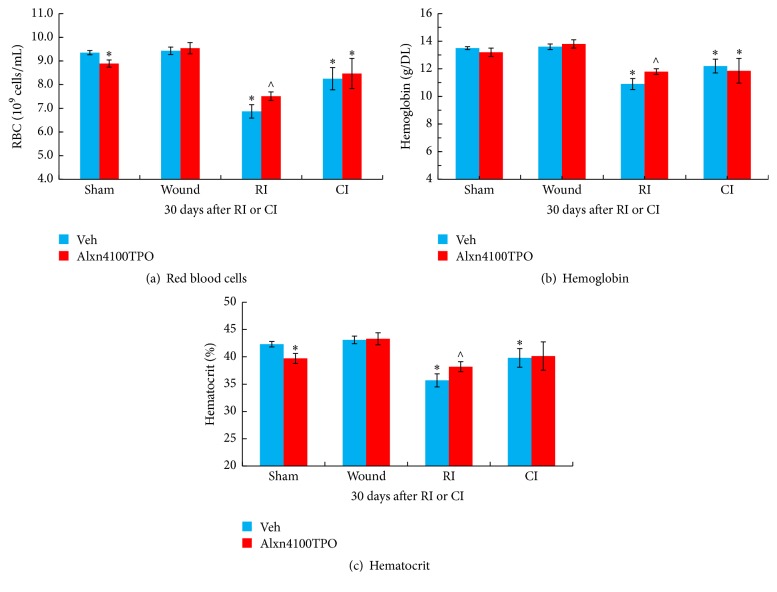
RI and CI decreased RBC counts, hemoglobin concentrations, and hematocrit values but Alxn4100TPO significantly mitigated only the RI-induced decreases. RBC counts, hemoglobin concentrations, and hematocrit values in blood samples collected 30 days after RI and CI were measured. *N* = 6 per group. Data are presented as mean ± SEM. ^*∗*^*p* < 0.05 versus Vehicle Sham; ^∧^*p* < 0.05 versus Vehicle RI. RI: 9.5 Gy; CI: 9.5 Gy and skin wound.

RI and CI were previously shown to reduce numbers of RBC, hemoglobin concentrations, and hematocrit values, whereas wounding alone did not alter these parameters [1]. In this present study these data in vehicle-treated mice surviving after RI and CI confirmed the observation (Figure 4). Treatment with Alxn4100TPO significantly mitigated only the RI-induced reductions of RBC numbers (Figure 4(a)), hemoglobin concentrations (Figure 4(b)), and hematocrit values (Figure 4(c)) compared to vehicle-treated mice. This treatment significantly decreased RBC numbers and hematocrit values in sham mice as well (Figure 4).

### 3.4. RI Induces Splenomegaly and Alxn4100TPO Mitigates It after RI but Induces It after CI

**Figure 5:**
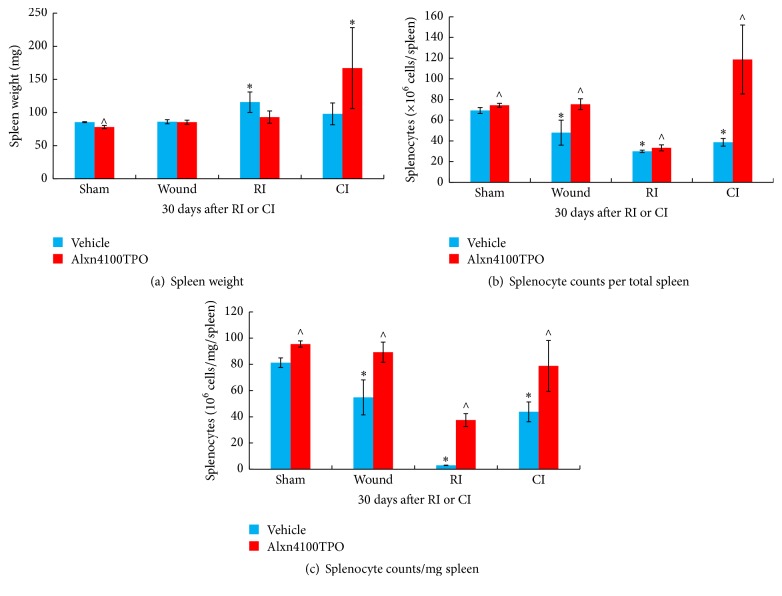
Different effects of Alxn4100TPO on spleen after RI or CI. (a) RI induced splenomegaly and Alxn4100TPO significantly mitigated splenomegaly but induced it after CI. (b) Wounding, RI, and CI decreased splenocyte numbers and Alxn4100TPO significantly increased them after sham, wounding, RI, and CI. (c) Each splenocyte count was normalized by its spleen weight. Spleens collected 30 days after RI and CI were weighed and then they were processed in a method to obtain resuspended cells. The numbers of splenocytes were counted. *N* = 6 per group. Data are presented as mean ± SEM. ^*∗*^*p* < 0.05 versus Vehicle Sham; ^∧^*p* < 0.05 versus respective vehicle group. RI: 9.5 Gy; CI: 9.5 Gy and skin wound.

RI is known to induce splenomegaly, whereas CI does not induce it [9, 10]. In this study, a similar result was observed 30 days after RI and CI in surviving mice (Figure 5(a)). Alxn4100TPO treatment inhibited RI-induced splenomegaly, but it tended to increase it after CI, although it was not statistically significant. The Alxn4100TPO treatment did not alter the spleen weight in sham and wounded mice (Figure 5(a)).

### 3.5. Wounding, RI, and CI Decrease Splenocyte Numbers and Alxn4100TPO Increases Splenocyte Numbers after Sham, Wounding, RI, and CI

Numbers of splenocytes were counted in each suspension of spleen cells. Wounding, RI, and CI significantly reduced numbers of splenocytes 30 days later in these surviving mice. Alxn4100TPO treatment increased splenocytes in each spleen of sham, wounded, surviving RI, and surviving CI mice (Figure 5(b)). Similar observations were obtained when each splenocyte count was normalized with each individual spleen weight (Figure 5(c)).

### 3.6. CI Further Decreases RI-Induced Survival and Alxn4100TPO Improves Survival in CI Mice

**Figure 6:**
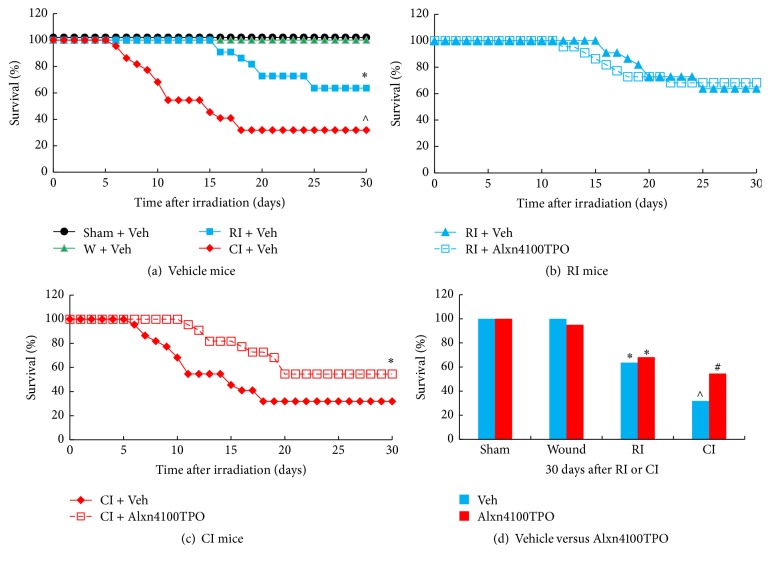
RI and CI decreased survival and Alxn4100TPO improved survival after CI. Animals were monitored for 30 days. *N* = 19–22 per group. For panel (a): ^*∗*^*p* < 0.05 versus sham and W; ^∧^*p* < 0.05 versus RI. For panel (c): ^*∗*^*p* < 0.05 versus CI + Vehicle. For panel (d): ^*∗*^*p* < 0.05 versus sham and W; ^∧^*p* < 0.05 versus RI + Vehicle; ^#^*p* < 0.05 versus CI + vehicle. W: wound; RI: 9.5 Gy; CI: 9.5 Gy and skin wound.

CI decreased survival to 31% (*p* < 0.05, *N* = 22), which was less than the survival observed in RI mice (65%, *p* < 0.05, *N* = 22) (Figures 6(a) and 6(d)). In CI mice, treatment with Alxn4100TPO increased survival by a 23-percentage differential after CI (Figures 6(c) and 6(d), *p* < 0.05) but in RI mice, treatment with Alxn4100TPO failed to alter 30-day survival (Figures 6(b) and 6(d), *p* > 0.05). Survival was 100% in mice that were given skin wounding alone (*N* = 19) but 1 mouse died out of 20 that were treated with Alxn4100TPO during the 30-day observation period due to septic infection (Figure 6(d)).

### 3.7. CI Further Increases RI-Induced Body Weight Loss and Alxn4100TPO Mitigates the Loss

**Figure 7:**
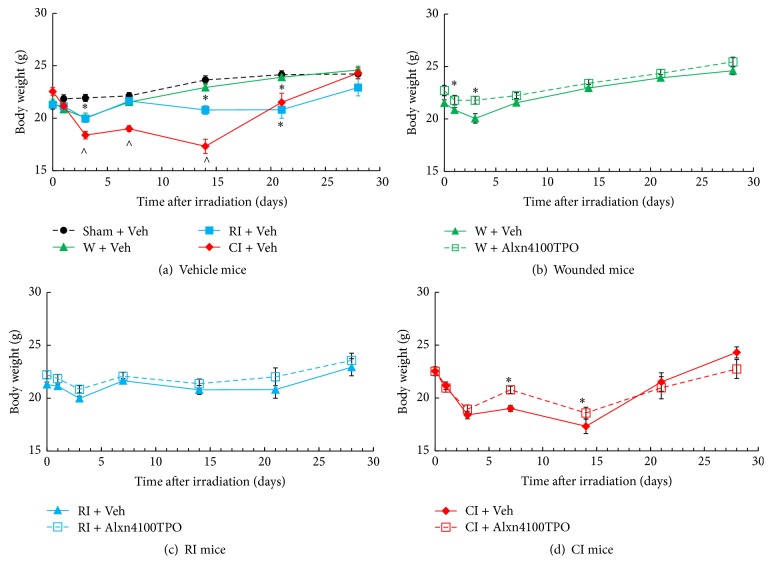
RI and CI decreased body weight and Alxn4100TPO significantly improved body-weight loss after CI. Body weights of animals were measured on days 1, 3, 7, 14, 21, and 28. *N* = 19–22 per group. Data are presented as mean ± sem. For panel (a): ^*∗*^*p* < 0.05 versus sham, W, and CI; ^∧^*p* < 0.05 versus sham, W, and RI. For panel (d): ^*∗*^*p* < 0.05 versus CI + Vehicle. Veh: vehicle; W: wound; RI: 9.5 Gy; CI: 9.5 Gy and skin wound.

RI is known to reduce the body weight starting on day 2 after RI and CI [1]. Therefore, body weights were measured six times during the 30-day experimental period. We observed that skin wounding alone induced body-weight loss on day 1 and day 3 and that Alxn4100TPO treatment inhibited the loss (Figure 7(b)). RI decreased body weights, but Alxn4100TPO was ineffective in blocking the loss (Figure 7(c)). CI enhanced the RI-induced body-weight loss beginning on day 3 and up to day 14 after CI (Figure 7(a)). Treatment with Alxn4100TPO mitigated body-weight loss in CI mice (Figure 7(d)) on days 7 and 14 and recovered body weight equivalent to controls by day 28 (Figure 7(d)).

### 3.8. CI Enhances Water Consumption and Alxn4100TPO Decreases the Enhanced Water Consumption

**Figure 8:**
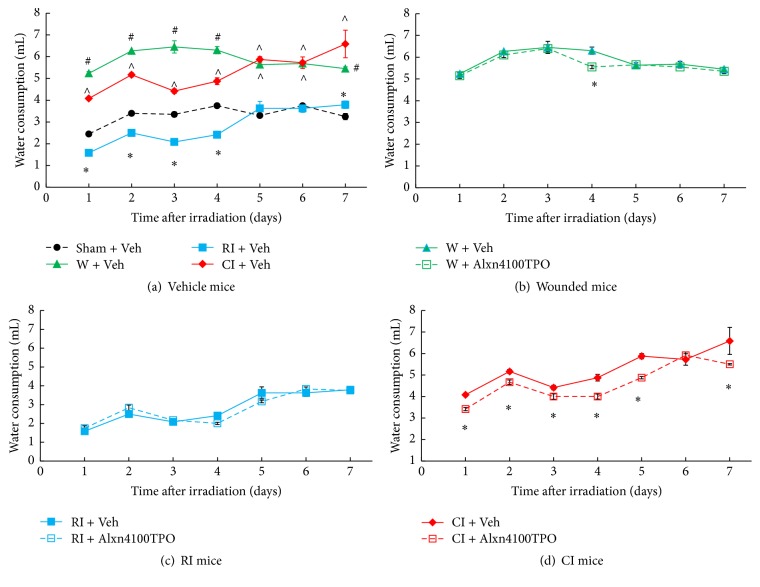
RI decreased and CI increased water consumption and Alxn4100TPO significantly decreased the CI-enhanced water consumption. The daily water consumption was monitored for 7 days after wounding, RI, or CI. *N* = 19–22 per group. Data are presented as mean ± SEM. For panel (a): ^*∗*^*p* < 0.05 versus sham, wound, and CI; ^∧^*p* < 0.05 versus sham, wound, and RI; ^#^*p* < 0.05 versus sham, RI, and CI. For panel (b), ^*∗*^*p* < 0.05 versus W + Vehicle. For panel (d): ^*∗*^*p* < 0.05 versus CI + Vehicle. Veh: vehicle; W: wound; RI: 9.5 Gy; CI: 9.5 Gy and skin wound.

It is evident that, in comparison to the sham group, wounding alone significantly increased water consumption since day 1 after wounding presumably because wounded mice required more water to maintain proper hydration to replace the loss of body moisture through the open wound. In contrast, RI mice reduced water consumption below that in sham mice during the first four days. Water consumption was reduced in CI mice below that in wounded mice but above that in sham mice (Figure 8(a)). Treatment with Alxn4100TPO reduced water consumption in CI mice on days 1–5 and 7 (Figure 8(d)) and in wounded mice slightly on day 4 (Figure 8(b)), but Alxn4100TPO did not change RI-induced reduction in water consumption (Figure 8(c)).

### 3.9. CI Delays Wound-Healing Ability and Alxn4100TPO Increases the Delay

**Figure 9:**
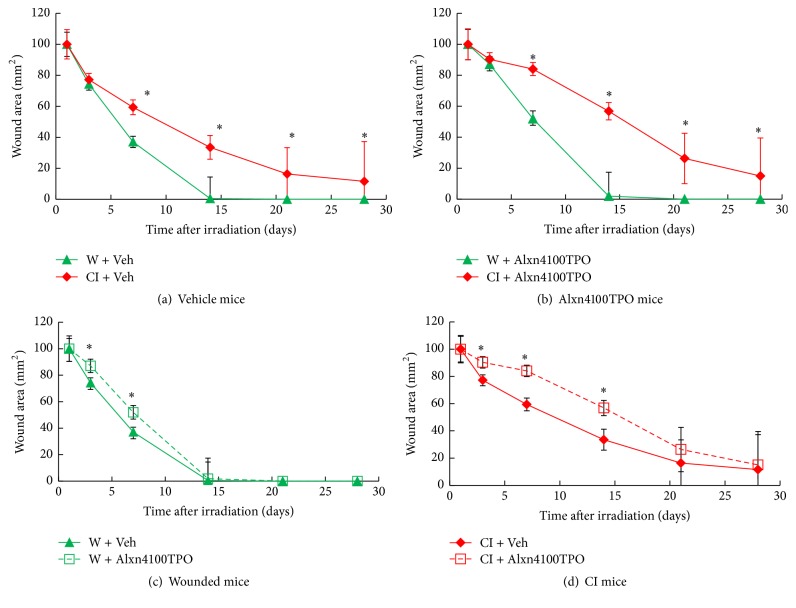
CI delays wound healing, but Alxn4100TPO increased the delay. Wound areas were measured on days 1, 3, 7, 14, 21, and 28. *N* = 19–22 per group. Data are presented as mean ± SEM. For panel (a): ^*∗*^*p* < 0.05 versus RI. For panel (b), ^*∗*^*p* < 0.05 versus W + Alxn4100TPO. For panel (c): ^*∗*^*p* < 0.05 versus RI + Vehicle. For panel (d): ^*∗*^*p* < 0.05 versus CI + Vehicle. Veh: vehicle; W: wound; RI: 9.5 Gy; CI: 9.5 Gy and skin wound.

It is evident that it takes 14 days to have full wound healing in non-irradiated wounded mice, but RI delayed wound-healing rates by an additional 14 days among survivors (Figure 9(a)). Treatment with Alxn4100TPO delayed the healing rate slightly in wounded mice at days 3 and 7 and increased the delay in CI mice through day 21 (Figures 9(b)–9(d)).

## 4. Discussion

A single s.c. dose of Alxn4100TPO 24 h after irradiation improved survival by a 23-percentage differential in CI mice but not in RI mice (Figure 6). Treatment with Alxn4100TPO also significantly increased the numbers of platelets (Figure 2) and splenocytes (Figure 5) in surviving mice 30 days after RI and CI. This platelet result agrees with a previous observation [13]. Perhaps, one of the principal reasons that Alxn4100TPO improves the survival rate in CI mice is by promoting recovery of platelets, which, in turn, prevent internal bleeding by restoring the clotting mechanism. Moreover, Alxn4100TPO induced increases in spleen weights and splenocytes that consist of a variety of cell populations such as T and B lymphocytes, dendritic cells, and macrophages, which have different immune functions. We are not clear which types of cells in spleen got increased. Since megakaryocytes are known to be produced in spleen, a histological slide examination can evaluate whether Alxn4100TPO treatment increased megakaryocyte counts in spleen of CI mice. The information will be useful.

Alxn4100TPO therapy did not improve survival after RI in our study, which is inconsistent with data observed in other laboratory [13]. Perhaps the B6D2F1 female mouse studied here responds differently than the CD2F1 male mice used in another study [13] due to the gender difference and the strain difference. It is also possible that higher doses of Alxn4100TPO with multiple injections are needed to upregulate neutrophil counts and subsequently improve the survival after RI in B6D2F1 female mice. Therefore, this drug needs to be studied further in RI mice.

It is evident that recombinant human TPO (rHuTPO) treatment (25 *μ*g/kg, s.c., +24 h once daily for 7 days) increased the survival rate in adult male C57BL/6 after lethal irradiation [19]. It is of interest to study whether rHuTPO treatment can increase survival in B6D2F1 female mice after both RI and CI. Nonetheless, the optimal Alxn4100TPO administration protocol and its drug reduction factor should be determined at LD_30/30_, LD_50/30_, and LD_70/30_ in RI mice and CI mice. Therefore, more studies are warranted to be conducted.

This report confirms in vehicle groups that radiation decreased survival, increased body-weight loss, and decreased water consumption and that combined injury decreased survival and body weight significantly but elevated water consumption more than radiation alone in B6D2F1/J mice. Radiation delayed wound-healing abilities. These results are consistent with previous observations in rats [20, 21], guinea pigs [22], dogs [23], swine [24], and mice [1, 5, 6, 15, 25–27]. Medical sequelae of either RI or CI include acute myelosuppression, immune system inhibition, fluid imbalance, systemic inflammation, macro-/microcirculation failure, massive cellular damage, sepsis, and disruption of vital organ functions, which lead to MOD and MOF, the most frequent causes of death after irradiation [22–24]. The increased mortality after CI more than RI is thought to be attributed by the magnified NF-*κ*B/NF-IL6/iNOS signaling, cytokine concentrations, and severe sepsis [7]. Similar results are observed after irradiation combined with hemorrhage or burn [6, 9, 10, 28].

Drugs such as 5-androstenediol [29], G-CSF [9], and captopril [30] have been noted to be effective for mitigating RI-induced lethality; however, when these drugs were administered for CI treatment, they were ineffective in improving survival after CI, implying that skin wound trauma requires additional remedies specific for wound treatment. Thus, we continue to pursue evaluation of drugs that could alleviate sequelae of CI, such as Alxn4100TPO, because it is a TPO receptor agonist and increases numbers of platelets and survival after RI [13] and TPO is a major physiological regulator of platelet production [31]. It attenuates the thrombocytopenia related to myelosuppressive chemotherapy or radiation-induced injury [32–37]. The data of Alxn4100TPO-induced increases in RBCs in normal mice or after RI is in agreement with that reported in another laboratory [34, 36, 37].

Alxn4100TPO therapy reduced water consumption in CI mice, which required additional water to rehydrate. This effect would be expected to decrease survival, yet, the drug also improved survival in CI mice. This is quite an enigma. Why would a substance which deprives the mice of water that they need to survive at the same time supports their survival? Apparently, beneficial effects of Alxn4100TPO, which improve survival, compensate for some proportional decrease in water consumption.

In surviving CI mice but not in surviving RI mice on day 30, Alxn4100TPO therapy also increased WBC counts (approx. 1.61 × 10^6^ cells/mL), mainly by increased numbers of neutrophils (approx. 0.7 × 10^6^ cells/mL), lymphocytes (approx. 0.38 × 10^6^ cells/mL), monocytes (approx. 0.09 × 10^6^ cells/mL), and eosinophils (approx. 0.34 × 10^6^ cells/mL). Increase in numbers of neutrophils in particular could improve ability of a mouse to control bacteremia or even alleviate sepsis, thus improving survival. The drug-induced increase in WBC counts only in CI mice suggests the possible involvement of an unidentified mechanism and warrants further investigation. RI and CI, however, significantly reduced WBC counts (Figure 3), in agreement with previous observations [1, 2]. At day 30 after RI or CI, surviving mice still displayed low WBC counts, mainly lymphocytes, monocytes, and eosinophils, and basophils after RI and lymphocytes, eosinophils, and basophils after CI (Figure 3). However, CI-induced decreases were significantly mitigated only in surviving Alxn4100TPO-treated CI mice, whereas RI-induced decreases were not mitigated by this drug.

Alxn4100TPO increased RBC counts, hemoglobin levels, and hematocrit readings in RI mice but not in CI mice, suggesting that this drug may activate erythropoietin in RI mice. This view is further reinforced by Alxn4100TPO's inhibition of RI-induced splenomegaly, because splenomegaly is a result of large volume of dead RBCs. But Alxn4100TPO increased splenomegaly in CI mice, suggesting that the increase may be beneficial towards the animal's well-being and subsequently increasing survival after CI. Nevertheless, the risk of thrombosis due to significant increases in circulatory platelets cannot be excluded. Therefore, delayed effects of Alxn4100TPO in these surviving CI mice should be followed for another 120 days to monitor the risk of thrombosis.

On the other hand, RI-induced decreases in RBC counts, hemoglobin, and hematocrit values were mitigated only in surviving Alxn4100TPO-treated RI mice, whereas CI-induced decreases were not. These results suggest that the mitigation of either WBC or RBC are at least partially essential for mouse survival. It seems that some other not yet identified factor that is not conferred by Alxn4100TPO is crucial for survival after CI. However, it should bear in mind that determining Alxn4100TPO blood levels at different time points would provide therapy-relevant pharmacokinetic information.

We speculate that wound healing probably plays a key role in determining the turning point between life and death after CI. Although Alxn4100TPO therapy increased survival rate, it did not accelerate wound-healing rate after CI (Figure 9). The deficiency in wound-healing acceleration could be one of the key factors that limit the efficacy of this drug for survival improvement after CI, because drugs such as ghrelin [9] or ciprofloxacin [38–41] or stem cell therapy [41–43], which significantly accelerates wound healing, greatly improved survival after CI. Therefore, we postulate that an ideal single drug that could mitigate H-ARS as well as cutaneous syndrome could be a minimum requirement to mitigate injury after both RI and CI. Another option is to give a combined therapy of at least two drugs with one for treating H-ARS and the other for treating cutaneous syndrome. The last option but not the least is a drug/agent/substance that can store or maintain water within the bodies leading to mitigation of body weight loss after RI and CI.

In summary, Alxn4100TPO therapy improved 30-day survival and significantly mitigated body-weight loss, mitigated WBC depletion, and elevated platelets and splenocytes after CI but delayed the wound-healing rate. Alxn4100TPO mitigated the RI-induced decreases in RBC counts, hemoglobin levels, hematocrit values, platelet counts, and splenocyte counts but failed to improve body-weight loss and survival after RI. These results demonstrate a limited efficacy of Alxn4100TPO therapy for CI. However, Alxn4100TPO might provide a component of a multidrug therapeutic regimen for CI, particularly since it increased circulating platelet numbers, which would reduce internal bleeding, and elevated splenocyte numbers, which would increase immune function to fight away infection that usually occurs after RI and CI.

## Abbreviations

AFRRI: Armed Forces Radiobiology Research Institute
ARS: Acute radiation syndrome
CI: Combined injury
^60^Co: Cobalt-60
ESR: Electro Spin Resonance
G-CSF: Granulate colony stimulating factor
Gy: Gray
H-ARS: Hematopoietic component of acute radiation syndrome
IL: Interleukin
iNOS: Inducible nitric oxide synthase
KC: Keratinocyte factor
LD_30/30_: Lethal dose for 30% of population dead within 30 days
LD_50/30_: Lethal dose for 50% of population dead within 30 days
LD_70/30_: Lethal dose for 70% of population dead within 30 days
MCP-1: Monocyte Chemoattractant Protein-1
MOD: Multiorgan dysfunction
MOF: Multiorgan failure
NF-*κ*B: Nuclear factor-kappa B
NF-IL6: Nuclear factor-interleukin 6
p.o.: Per os (*L*., by mouth, orally)
RBC: Red blood cell
RCI: Radiation combined injury
RI: Radiation injury
s.c.: Subcutaneous or subcutaneously
TBSA: Total body surface area
TPO: Thrombopoietin
VSD: Veterinary Science Department
W: Wound
WBC: White blood cell.
